# Essential Roles and Risks of G-Quadruplex Regulation: Recognition Targets of ALS-Linked TDP-43 and FUS

**DOI:** 10.3389/fmolb.2022.957502

**Published:** 2022-07-11

**Authors:** Akira Ishiguro, Akira Ishihama

**Affiliations:** Research Center for Micro-Nano Technology, Hosei University, Tokyo, Japan

**Keywords:** G-quadruplex (G4), amyotrophic lateral sclerosis (ALS), liquid-liquid phase separation (LLPS), RNP granule, TDP-43 (43 kDa TAR DNA-binding protein), FUS (fused in sarcoma)

## Abstract

A non-canonical DNA/RNA structure, G-quadruplex (G4), is a unique structure formed by two or more guanine quartets, which associate through Hoogsteen hydrogen bonding leading to form a square planar arrangement. A set of RNA-binding proteins specifically recognize G4 structures and play certain unique physiological roles. These G4-binding proteins form ribonucleoprotein (RNP) through a physicochemical phenomenon called liquid-liquid phase separation (LLPS). G4-containing RNP granules are identified in both prokaryotes and eukaryotes, but extensive studies have been performed in eukaryotes. We have been involved in analyses of the roles of G4-containing RNAs recognized by two G4-RNA-binding proteins, TDP-43 and FUS, which both are the amyotrophic lateral sclerosis (ALS) causative gene products. These RNA-binding proteins play the essential roles in both G4 recognition and LLPS, but they also carry the risk of agglutination. The biological significance of G4-binding proteins is controlled through unique 3D structure of G4, of which the risk of conformational stability is influenced by environmental conditions such as monovalent metals and guanine oxidation.

## Introduction

**FIGURE 1:**
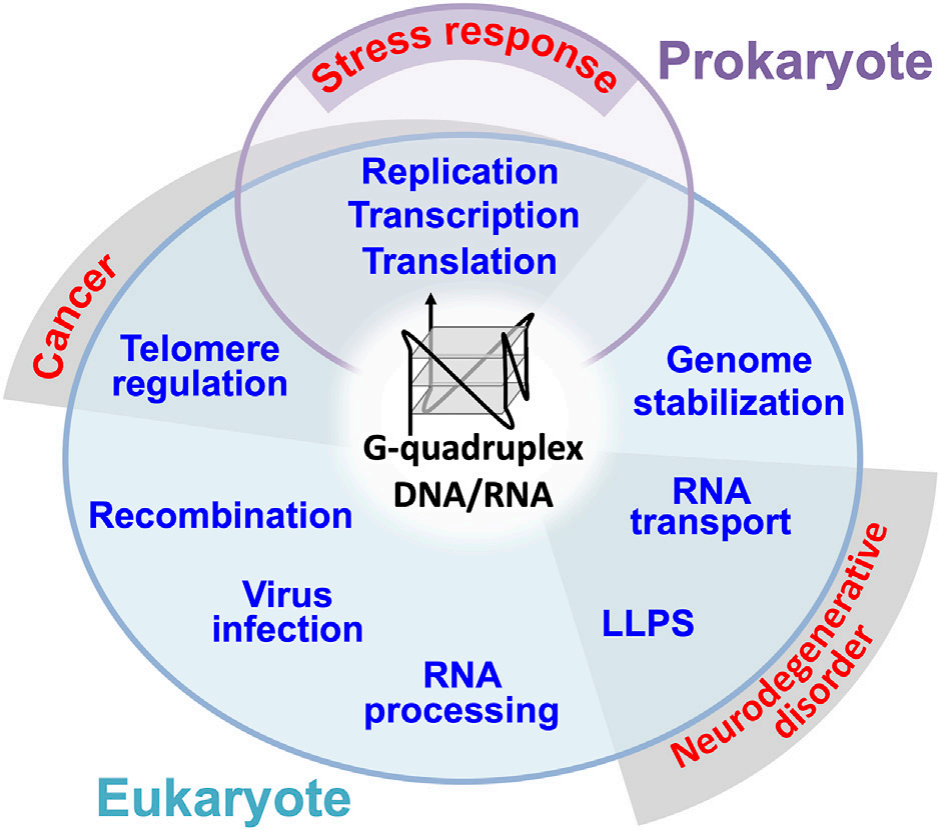
General roles of DNA/RNA G4s in prokaryote and eukaryote. G4 has also been suggested to be involved in prokaryotic stress responses through replication, transcription, and translation. Dysregulation of telomere regulation, transcription, translation and replication by G4 are suggested to be involved in the pathogenic mechanism of human cancer. Mutations in G4 containing RNAs and G4 binding proteins involved in RNA transport and LLPS have been found in patients with neurodegenerative diseases such as ALS.

A non-canonical DNA/RNA structure, G-quadruplex (G4) is formed by two or more guanine quartets, which associate through Hoogsteen base pairs leading to form a square planar arrangement stabilized by a central cation (Rodes and Lipps, 2015). The G4 structure first discovered *in vitro* was found to be telomere-formed structures, and later abundant G4-foming sequences have been identified in the genome (Sen and Gilbert, 1988, Sundquist and Klug, 1989; Rodes and Lipps, 2015). Such G4-forming sequences are present in both prokaryotes and eukaryotes. For instance, a total of 52 G4 motifs are present in the *E. coli* genome while more than 700,000 G4 motifs are present in the human genome (Métifiot et al., 2014; Doluca et al., 2016; Kaplan et al., 2016; Marsico et al., 2019; Ruggiero and Richter, 2020; Tu et al., 2021). Some of these G4 motifs are involved in DNA replication, recombination, telomere regulation and transcriptional regulation (Figure 1) (Métifiot et al., 2014; Yang, 2019; Prorok et al., 2019; Cammas and Millevoi, 2017). Some G4 motifs are copied into RNAs, thereby leading to the regulation of RNA functions such as processing, stability, translation and transport (Figure 1) (Bugaut and Balasubramanian, 2012; Cammas and Millevoi, 2017; Yang, 2019; Shao et al., 2020; Dumas et al., 2021; Georgakopoulos-Soares et al., 2022). G4-RNAs are often assembled into unique ribonucleoprotein (RNP) granules without boundary membranes, which were identified as precipitats in the brain tissue and cultured cell extracts using biotinylated isoxazole (Subramanian et al., 2011; Han et al., 2012). The membraneless RNP organelles formed through liquid-liquid phase separation (LLPS) are classified into stress granule, germ granule, P-bodie, neuronal granule, nuclear puncta, RNA-transport granule, RNA foci, and paraspeckle (Rhine et al., 2020). It has been suggested that the G4 structure supports the formation of condensates (Rhine et al., 2020; Roden and Gladfelter, 2021), which is the opposite to the inhibition of condensation by promiscuous RNA assembly (Maharana et al., 2018). Eventually, in 2021, four laboratories experimentally demonstrated that G4 promotes the condensation of DNA/RNA-protein complexes through LLPS (DNA: Liu et al., 2021; Gao et al., 2021; DNA: Mimura et al., 2021; RNA: Ishiguro et al., 2021).

G4s and G4-binding proteins in eukaryotes are presumed to exhibit the stress response and pathologically related functions including cancer and neurodegenerative disorder (see Figure 1). Therefore, G4s and G4-binding proteins could be either the potential protection targets or pathogenic drivers in the pathogenic cascade of various diseases including neurological disorders (Simone et al., 2015; Wang et al., 2021). The amyotrophic lateral sclerosis (ALS), a neurodegenerative disease, is a multifunctional disorder involving both genetic and non-genetic risk factors (Taylor et al., 2016; Deng et al., 2017; Scarlino et al., 2020). Genes encoding many RNA-binding proteins have been identified for genetic risks, and recent studies suggest their involvement in G4 and RNP granules as a common function. In this review article, we summarize the roles and risks of G4-contaning RNAs and two G4-RNA binding proteins, TDP-43 (43 kDa TAR DNA-binding protein) and FUS (fused in sarcoma).

## Dedicated Methods of G4-RNA

Research on molecular mechanisms of G4s and G4 binding proteins is still in its infancy stage. In fact, some RNA binding proteins have recently been re-identified one after another as G4-RNA binding proteins (Byrd et al., 2016; Ishiguro et al., 2016; Ramesh et al., 2020; Simko et al., 2020; Zheng et al., 2020; He et al., 2021; Ishiguro et al., 2021). Attempts to identify bound proteins with known G4s have been performed by mass spectrometry and proteome microarray (Mori et al., 2013; Zhang et al., 2021). On the other hand, however, attempts to identify binding target RNAs from the protein side are often difficult because of technical problems. Thus, the correct choice of analytical methods for identification of G4-RNAs could be the driving force for discovering unidentified G4-RNA regulatory systems.

Identification of non-canonical G4 motifs is different from that of canonical RNA sequences (Guo and Bartel, 2016; Yang et al., 2020). For example, cross-linking and immunoprecipitation (CLIP), one of the most used methods for identifying RNAs as protein binding targets from whole cellular RNA, often result in selective loss of most non-canonical G4 motifs. Unlike Watson-Click pairing, the high stability of G4 structure due to extensive hydrogen-bonding and base-stacking interactions interfere with reverse transcription and PCR in the process (Guo and Bartel, 2016; Stevens et al., 2017). Therefore, several methods have been developed such as rG4-seq (Guo and Bartel, 2016; Kwok et al., 2016; Yang et al., 220) and ADO (allelic drop) (Stevens et al., 2017). Due to incomplete identification of in the high-throughput screening, it is necessary to confirm the prediction by biochemical analysis of direct interactions between the proteins and the target RNA candidates using such as the gel shift assay. However, the sharply bent DNA/RNA-protein complexes do not migrate normally in native acrylamide gel electrophoresis and G4-protein complex often retained in the top of gel, if the complex is correctly formed (Carey et al., 2010; Al-Furoukh et al., 2013; Ariyo et al., 2015; Huang et al., 2017; Mou et al., 2022). Furthermore, it is difficult to increase the concentration of monocations in the gel during electrophoretic fractionation, which is necessary for the stabilization of G4 tetrads. thus often leading to incorrect identification (Yakhnin et al., 2012; Kim et al., 2015). To avoid these problems, use in combination with other methods such as the filter-binding assay, thermophoresis assay or surface plasmon resonance analysis is effective to determine the specific interaction between G4-RNA and G4-RNA binding protein (von Hacht et al., 2014; Ishiguro et al., 2016; Mou et al., 2022).

## G4-RNAs and G4-DNAs in Prokaryote

G4-forming sequence in bacteria genome has often been identified in the promoter region (Dey et al., 2021; Yadav et al., 2021), suggesting the involvement of G4 in transcription regulation (see Figure 1). In fact, the expression of some stress response genes such as radioprotection-related genes are under the control of G4-containing promoters (Beaume et al., 2013; Kumari and Raghavan, 2021). Roles for G4s have also been posited in the antigenic variation systems of bacteria (Harris and Merrick, 2015). Along this line, G4 could be used for the development of antimicrobial target (Yadav et al., 2021). G4 is also suggested in the maintenance of bacterial genome. For instance, *E. coli* Rep helicase and RecA recombinase unwind the G4 DNA, thus stabilizing toleration of toxicity induced by G4-stabilizing ligands (Paul et al., 2020; Xue et al., 2020). As in the case of eukaryote, *E. coli* RNA polymerase forms clusters, together with transcription anti-terminator NusA, LLPS-mediated biomolecular condensates, (Ladouceur et al., 2020). However, the biological relevance of G4 DNA/RNA in prokaryotes has only begun to emerge even though their very important and conserved biological functions.

## G4-RNAs and G4-Binding RNA Proteins as the Genetic Causes of Amyotrophic Lateral Sclerosis

As to the G4-RNAs and G4-RNA binding proteins in eukaryote, the well-analyzed systems are those involved in ALS, the neurodegenerative disorder characterized by the progressive death of upper and lower motor neurons (Taylor et al., 2016) (see Figure 1). Approximately 90% cases of ALS are called sporadic, and a history of ALS has been identified for only about 10% of familial cases (Renton et al., 2014; Scarlino et al., 2020). The peak age of ALS onset is 58–63* *years for sporadic, and 47–52* *years for familial (Kiernan et al., 2011). More than 50 ALS-associated genes have been identified, of which gain or loss of the gene function led to the risk (Mejzini et al., 2019). These gene products are involved in various cellular processes such as redox regulation, signal transduction, and axonal transport of RNAs. The most abundant gene species encode RNAs and RNA-binding proteins, such as intronic expansion of the human *C9orf72* (chromosome 9 open reading frame 72) gene, TDP-43, FUS, hnRNPA1 (heterogeneous nuclear ribonucleoprotein A1), hnRNP A2/B1, hnRNPA3, EWSR1 (Ewing’s sarcoma RNA binding protein 1), and TIA1 (T cell-restricted intracellular antigen-1) (Taylor et al., 2016; Ishiguro et al., 2021). Surprisingly, these RNA binding-proteins recognize and bind to mRNA containing G4 motifs (Ishiguro et al., 2021). For instance, ALS-linked intronic expansion of the human *C9orf72* gene and potentially causative two long noncoding RNAs, NEAT1 (nuclear enriched abundant transcript 1) and MALAT1 (metastasis associated in lung adenocarcinoma transcript 1/NEAT2) contain abundant G4 motifs (Grigg et al., 2014; Haeusler et al., 2014; Simko et al., 2020; Mou et al., 2022). More interestingly, these ALS-related proteins/RNAs constitute RNP granules or regulate the formation of RNP granules (Tourrière et al., 2003; Fujii et al., 2005; Guil et al., 2006; Bosco et al., 2010; Molliex et al., 2015; Mensch et al., 2018; Ishiguro et al., 2021). RNP granules assembled by liquid-liquid phase separation (LLPS) are in turn involved in the regulation of the functions of many RNAs (Freibaum et al., 2021). RNP granules can also control the long-distance transport of mRNA, local translation, and mRNA silencing under stress conditions (Freibaum et al., 2021). Dysregulation of the system might contribute to the development and progression of ALS (Rotem et al., 2017; Gamarra et al., 2021; Milicevic et al., 2022).

## A Structural Feature Common to G4 Recognition and Liquid-Liquid Phase Separation

The G4-binding proteins often carry one or more intrinsically disordered regions (IDRs) (Lin et al., 2015; Molliex et al., 2015; Wheeler et al., 2016; Uversky, 2017; Martin and Holehouse, 2020). In generally, the interfaces of DNA/RNA-binding proteins are enriched in positively charged and aromatic residues, lacking negatively charged and Pro residues (Zhang et al., 2019; Bartas et al., 2021). These G4 RNA-binding proteins all have low complexity IDRs (Bartas et al., 2021; Dettori et al., 2021), which contain two to five different amino acids making up at least 50%, critically enriched in Glu, Ser, Lys, Pro, Gly, Ala, and Arg, but lacking aromatic residues (Dettori et al., 2021).

Intrinsically disordered proteins drive protein clustering and subsequent LLPS using their IDRs (Darling et al., 2018; Jo et al., 2021). These G4-RNA binding proteins form condensates mediated through LLPS. The LLPS formation can potentially be mediated by disorder-to-order transitions of dynamic IDR-RNA complexes. In addition, cation-π and π-π interactions have been suggested to regulate the phase behavior of constituent proteins in driving LLPS (Vernon et al., 2018; Paloni et al., 2020). G4 composed of guanine tetrads stacked by π-π interactions may provide more π-interactions for condensate formation (Raguseo et al., 2020; Ishiguro et al., 2021).

## Diversity of G4-RNA Binding Proteins: TDP-43 and FUS

**FIGURE 2:**
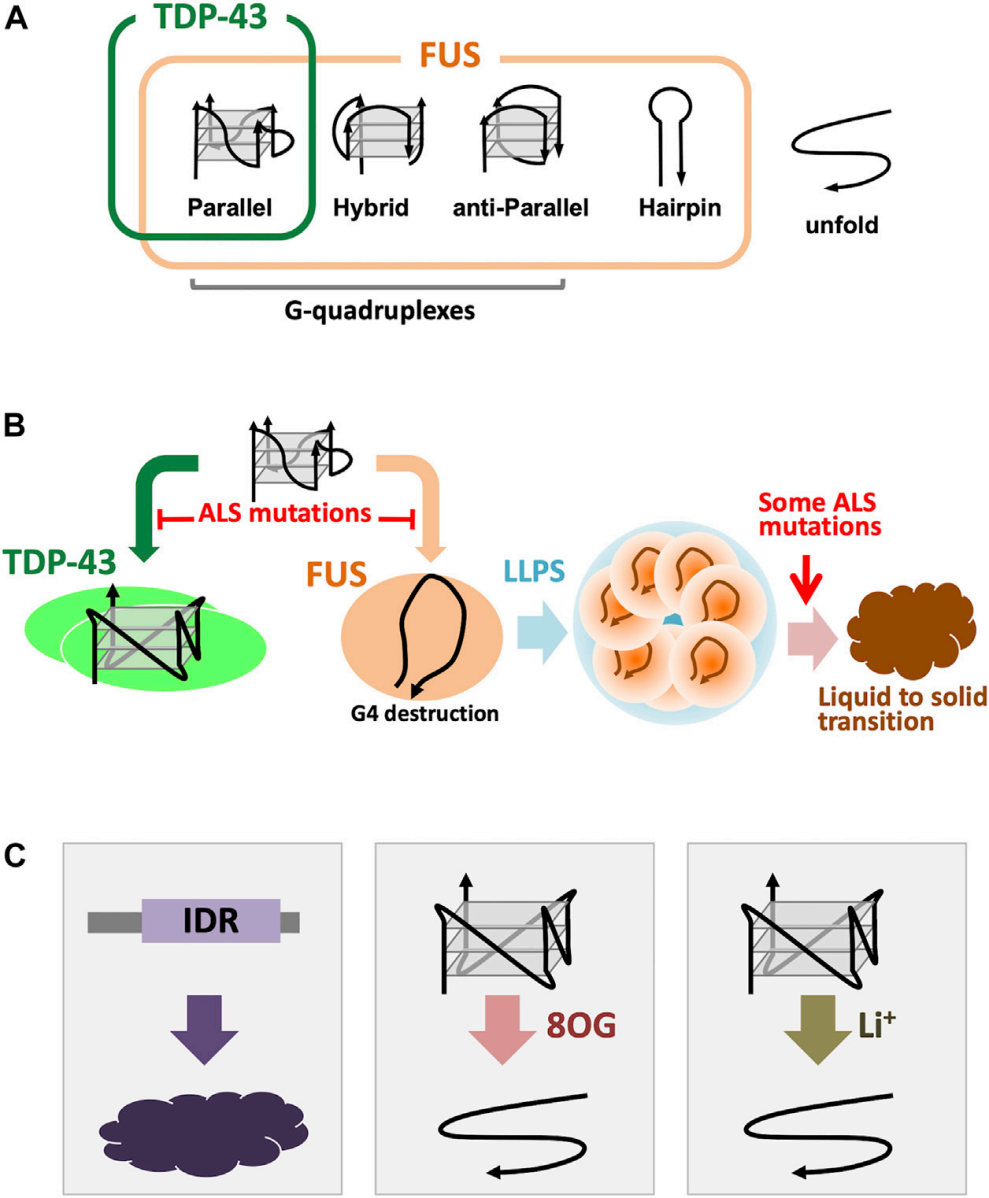
ALS-linked G4 binding proteins. **(A)**, Binding specificity of TDP-43 and FUS. TDP-43 can bind only for the parallel-stranded G4-DNA/RNAs, however, FUS binds to parallel, hybrid, and even G-rich hairpin forms (Ishiguro et al., 2021). **(B)**, Different G4 binding modes for TDP-43 and FUS. TDP-43 protects and stabilizes the G4 structure, while FUS destabilizes the G4 conformation and recreates it into a “terminal associated” configuration. In both cases, the G4-protein interaction was inhibited by introducing amino acid substitutions from ALS patients (Ishiguro et al., 2021). In some FUS mutations have been found that promotion of the phase transition of liquid to solid phase of the droplet formed by LLPS (Ishiguro et al., 2021). **(C)**, Concerned risks for G4s and G4 binding proteins. Low-complexity IDRs have considered to be tended to be aggregated (van der Lee et al., 2014; Taylor et al., 2016). 8OG produced by oxidation of guanine interferes with the normal formation of G4. Li+ is the same monocation as K+ and Na+, but it is known that it may not contribute to the structural stabilization of G4.

The best characterized ALS-linked G4-RNA binding proteins, TDP-43 and FUS, share structural and functional similarities, including participation in ALS pathogenesis (Lagier-Tourenne and Cleveland, 2009; da Cruz and Cleveland, 2011; Strong and Volkening, 2011; Guerrero et al., 2016; Ratti and Buratti, 2016; Ishiguro et al., 2021). RNP granules formed by TDP-43 and FUS play roles in axonal transport (Fujii et al., 2005; Subramanian et al., 2011; Alami et al., 2014; Ishiguro et al., 2016; Fay et al., 2017). Although both proteins recognize G4 structures, their binding specificity is different, thereby leading to different mode of action (Ishiguro et al., 2021). Three typical G4 topologies are known: parallel, anti-parallel and hybrid, of which most G4-RNAs carry the adopt parallel formation. TDP-43 binds only to the parallel-stranded G4 DNA/RNA (Figure 2A). The selective G4 recognition of TDP-43 to parallel stranded G4 is probably due to the dimerization of TDP-43, which has two RNA recognition motifs (RRMs) and a C-terminal Gly-rich IDR. The RRM fragment, and the full-length TDP-43 that does not form a homo dimer, exhibit binding to wide range of UG-rich sequences (Buratti and Baralle, 2001; Lukavsky et al., 2013; Rengifo-Gonzalez et al., 2021). Under physiological conditions, full-length TDP-43 forms a homodimer, which loses the binding priority to the UG-rich sequence, suggesting the presence of a complex binding mechanisms using multiple RNA-binding modules (Ishiguro et al., 2016; Ishiguro et al., 2021). In contrast, FUS shows binding to all three types of G4 and shows binding to G-rich hairpin structure (Loughlin et al., 2019; Ishiguro et al., 2021) (Figure 2A). We also found that TDP-43 induces stable G4 conformation of the target RNA, whereas FUS destabilizes G4 conformation into an open-structure with close association between 3′ and 5′ termini (Ishiguro et al., 2021) (Figure 2B).

The effects of amino acid substitution mutations from ALS patients are common to these two proteins, and TDP-43 and FUS mutant proteins show reduced interaction with G4. (Ishiguro et al., 2021; Ishiguro et al., 2021) (Figure 2B). ALS-linked FUS mutations induced altered formation of the G4-dependent droplets (Ishiguro et al., 2021) (Figure 2B). In addition, the phase transition from droplets to aggregates is also altered by FUS mutations (Figure 2B). These mutations are considered to result in the loss of function, but the possibility of a gain of function is yet not excluded.

## The Essential Risks for G4-RNAs and G4-RNA Binding Proteins

The assembly of RNP granules by G4-RNA and G4-RNA binding proteins control many biological processes, including mRNA transport from soma to distal neurites for local translation. However, there are several risks associated with the molecular properties of G4-RNA and G4-RNA binding proteins that lead to perturbation of the interplay between G4-RNA and G4-RNA binding protein including the pathogenesis of ALS. Several factors are shown below (Figure 2C):

### Protein Aggregation

ALS is characterized by the accumulation of protein aggregates in motor neurons (Blokhuis et al., 2013; Malik and Wiedau, 2020). However, it remains unsolved whether the aggregated proteins themselves are toxic due to gain of function or accompanied by loss of normal function. The presence of protein aggregation is, however, not a sufficient condition for the development and progression of ALS (Baloh, 2011; Blokhuis et al., 2013). G4 recognition and phase separation is mediated by low-complexity IDRs that are present in ALS-linked RNA binding proteins. The contribution of low-complexity IDR to those processes, however, carries the risk because IDRs intend to aggregate (van der Lee et al., 2014; Taylor et al., 2016).

### Guanine Oxidation

Oxidative stress is a risk factor of neurodegeneration and also a therapeutic target (Barber et al., 2006). Mutations of copper zinc superoxide dismutase 1 (SOD1) gene were found in 10%–20% of familial ALS cases (Rosen et al., 1993). Guanine has the lowest oxidation potential than other nucleobases and is readily oxidized to 8-oxoguanine (8OG), with elevated level of 8OG confirmed in the cervical cord of ALS patients (Chang et al., 2008). Cellular RNA is more susceptible target for guanine oxidation than DNA, and the oxidative damage to RNA could be a major focus in the investigation of neurodegenerative diseases (Hofer et al., 2005; Castellani et al., 2008). Since G4 is composed of many guanine bases, it should be sensitive to guanine oxidation. In fact, the insertion of 8OG into G4 motif causes structural destruction (Cheong et al., 2015).

### Lithium-Ion

G-quartets are stabilized in the presence of cations, but different cations result in different structural properties (Wang et al., 2014). Li^+^ is known as a factor that interferes with the uptake of Na^+^ and K^+^, which stabilizes the G-quadruplex structure *in vitro*. Although Na^+^ and K^+^ are the physiologically relevant monovalent ions in the context of G4 structures, however, Li^+^ with smaller ionic radius than Na^+^ and K^+^ causes destabilization of G4 conformation (Balaratnam and Basu, 2015; Bhattacharyya et al., 2016). Although induction of ALS due to extensive uptake of Li^+^ has not been observed, administration of lithium to ALS patients resulted in almost 70% of adverse events, including death (Chiò et al., 2010).

## Conclusion and Outlook

G4-associated regulations are involved in varieties of biological systems, including replication, recombination, telomere regulation, transcriptional regulation, RNA processing, RNA stability, translation, RNA transport, and stress responses. Although many of them have long been overlooked without experimental confirmation, but the advent of a new age arrived to reveal molecular details underlying the interaction of G4-RNAs and G4-binding proteins. The dysregulation of G4-RNAs and G4-binding proteins has been identified as neurodegenerative diseases in human. Risk factors in G4 and G4 binding proteins are involved in the pathogenic mechanism of ALS (for instance, Ishiguro et al., 2016; Ishiguro et al., 2020; Ishiguro et al., 2021). This review gives an overview on the interaction of G4-RNA with binding proteins and discussed dysregulation due to their structural properties, focusing the ALS-linked proteins and their interplay with G4-conteining RNAs. Unknown features affecting the risks along the interplay between G4-containing RNAs/DNAs and G4-binding proteins will be more clearly embodied in further investigation in this area.

As an extension of this line research, we have started to identify the role of G4 in the most well-characterized prokaryote *E. coli*. It may provide a platform for analyzing the formation of complex structures in G4.
